# Communication rehabilitation in sub-Saharan Africa: The role of speech and language therapists

**DOI:** 10.4102/ajod.v7i0.338

**Published:** 2018-04-12

**Authors:** Karen Wylie, Lindy McAllister, Bronwyn Davidson, Julie Marshall

**Affiliations:** 1 ENT Department, Korle Bu Teaching Hospital, Ghana; 2 Work Integrated Learning Department, Faculty of Health Sciences, University of Sydney, Australia; 3 Department of Audiology, Speech & Language Therapy, University of Ghana, Ghana; 4 Department of Audiology & Speech Pathology, The University of Melbourne, Australia; 5 Health Professions Department, Manchester Metropolitan University, United Kingdom

## Abstract

**Background:**

Workforce factors present a significant barrier to the development of rehabilitation services for people with communication disabilities in sub-Saharan Africa (SSA). Exploring how the work of speech and language therapists (SLTs) in the region is organised and delivered can provide insight into existing services, areas for future workforce development and improved rehabilitation access for people with communication disability.

**Objectives:**

This paper describes the employment and service provision patterns and work roles of a sample of SLTs in SSA.

**Method:**

A broad, purpose-designed, mixed-methods survey was designed to collect data from SLTs living in Anglophone countries of SSA. Descriptive statistics and qualitative content analysis were undertaken. This paper reports on a subset of data from the wider survey.

**Results:**

A description of the employment and work roles of the 33 respondents to the survey and characteristics of their service users is presented. SLTs were commonly employed within private and not-for-profit sectors and frequently worked in temporary jobs. SLTs engaged in a range of work roles, including capacity building and training others. Services were provided by SLTs across age ranges, health conditions and settings, with paediatric, urban services commonly reported. Costs for service users and urban-centred services give indications of barriers to service access.

**Conclusion:**

Knowledge of the way in which speech and language therapy services are organised and provided has the potential to shape the development of communication disability rehabilitation in SSA. This research has identified a range of issues requiring consideration as the profession develops and grows.

## Introduction

Despite an increasing global focus on inclusion and the rights of people with disabilities (PWD), rehabilitation services continue to be extremely limited in countries of the Majority World, including in sub-Saharan Africa (SSA). Many PWD report limited access to rehabilitation services (Eide & Loeb 2006; Eide & Kamaleri 2009; Loeb & Eide 2004; World Bank & World Health Organization 2011). Increasing the availability of rehabilitation and habilitation services for PWD is critical and forms one of the three objectives of the World Health Organization’s Disability Action Plan 2014–2021 (2015). (Note: In this paper the terms Majority and Minority World are used to replace the terminology ‘developed’ and ‘developing’ countries.) This paper focuses on workforce factors limiting the development of rehabilitation services for people with communication disability (PWCD) in SSA. PWCD have been described as:

a population whose ability to communicate is affected by their response to an impairment and/or social and contextual factors which interrelate with each other and with the person themselves, resulting in impaired communication skills. (Hartley 1998:277)

Two alternate paradigms have commonly been used to conceptualise disability. Historically, the medical model of disability was the dominant approach and considered impairment to be the causative factor in disability, while more recently the social model of disability attributed societal and environmental factors as the sole cause of disability. While debates about disability theory continue (Shakespeare & Watson 2002), in practice the biopsychosocial model of disability (World Health Organization 2001) has become widely adopted in rehabilitation. This model is represented in the International Classification of Functioning Disability and Health (World Health Organization 2001) and represents disability as a result of the inter-relationship between a health condition, personal and contextual factors, subsequently impacting a person’s activities and participation.

Responding effectively to the diverse rehabilitation needs of PWCD requires a workforce with a suitable mix of skills; however, there are recognised global shortages in the rehabilitation workforce, particularly in the Majority World (World Health Organization 2017). In SSA, speech and language therapists (SLTs) are rarely available (Fagan & Jacobs 2009) and community-based rehabilitation (CBR) workers frequently lack training in communication disability (Nganwa, Batesaki & Mallya 2013; World Bank & World Health Organization 2011). Indicative figures for the availability of SLTs in SSA are broadly suggestive of a workforce density of between 0 and 6 SLT per million population (Fagan & Jacobs 2009; Wylie et al. 2012). (Figures exclude South Africa where the profession of SLT is well established [Pillay & Kathard 2015].) However, there are indications of growth in the SLT profession in SSA across recent years, with the development of training programmes in a number of African countries, including Ghana, Togo, Kenya, Mozambique and Uganda (Wylie et al. 2016).

Currently, there is limited documented information about the nature and organisation of the work of SLTs in SSA. Understanding the characteristics of the existing speech and language therapy workforce, including the scope of practice, has potential to assist in the planning and strengthening of services (Gupta, Castillo-Laborde & Landry 2011) and allow consideration of how this emerging profession contributes to rehabilitation services for PWCD in the region. A previous paper (Wylie et al. 2016) described survey results from a sample of the SLT workforce in SSA, including their demographic composition, training and experience, and identified patterns suggestive of increasing localisation of the SLT workforce (i.e. more African nationals rather than foreigners working as SLTs). In light of the recent growth in training of SLTs (Wylie et al. 2016), there is an imperative to ensure the direction of the emerging profession and services are both appropriate and responsive to the needs in communities they serve. This paper contributes to the debate about the most appropriate way to develop services for communication disability rehabilitation in SSA by describing the employment patterns, roles and characteristics of service users of a sample of the SLT workforce in the region.

## Literature review

Many people with communication disabilities may seek rehabilitation across their lifetime. In this paper, the term rehabilitation is used to represent:

a set of measures that assist individuals who experience, or are likely to experience disability, to achieve and maintain optimal functioning in interaction with their environments. (World Bank & World Health Organization 2011:96)

Rehabilitation is cross-sectoral and may involve a wide range of workers in service delivery, including volunteers, CBR workers, allied health staff, doctors and family members. It is vital that an appropriate range of rehabilitation services are available, in order to promote participation in work, education and community engagement (World Health Organization 2015).

In the Majority World, including SSA, CBR services and medical rehabilitation services frequently coexist (World Health Organization 2015). Medical rehabilitation is often associated with health care systems, with services provided by professionals with skills in a particular area of rehabilitation (Haig 2013), including rehabilitation physicians, occupational therapists, physiotherapists and SLTs. In countries of the Minority World, medical rehabilitation services are frequently well developed. In contrast, CBR is widely adopted in Majority World including SSA (Hartley et al. 2010) and offers a broad approach to rehabilitation, using five interrelated components: health, education, livelihood, social and empowerment (International Labour Organization, UNESCO & World Health Organization 2010). CBR services are typically delivered by community workers with general and limited training. There is now increasing recognition that a range of rehabilitation approaches, including a mix of CBR and more specialised medical rehabilitation services, are essential to provide a holistic model of rehabilitation in the region (Nganwa et al. 2013). This includes increasing the availability of more specialised rehabilitation professionals, including SLTs (Nganwa et al. 2013; World Bank & World Health Organization 2011; World Health Organization 2017).

SLT is a Western profession that has evolved within the rehabilitation frameworks and belief systems of Europe and North America (Pillay & Kathard 2015; Sherry 2007) and is historically associated with medical rehabilitation. SLTs have specialised skills in the rehabilitation of communication and swallowing. In the Minority World, SLTs typically work with both children and adults with a range of communication and swallowing disabilities including primary communication disabilities such as speech and language delays or disorders, or those secondary to developmental disabilities, such as cerebral palsy, autism and hearing loss. Individuals may also seek communication rehabilitation services for acquired communication disabilities following a range of health events such as stroke, head injury or head and neck cancer. SLT services are commonly integrated within multidisciplinary rehabilitation teams in the Minority World countries.

SLTs, with their specialised knowledge in the field of communication disability, are likely to have key roles to play in communication disability rehabilitation in Majority World countries. These roles may include both providing communication disability rehabilitation for people with acute and complex communication needs and supporting and training CBR workers and others in providing basic rehabilitation services for PWCD (Wylie et al. 2016). However, it is unclear how SLTs currently work in Majority World contexts, where CBR and medical rehabilitation services may coexist.

Human resources for rehabilitation are a significant issue globally (Gupta et al. 2011; World Bank & World Health Organization 2011; World Health Organization 2017). One of the identified objectives of the WHO Disability Action Plan (2014–2021) (World Health Organization 2015) is to strengthen and extend rehabilitation services, through development and maintenance of a sustainable rehabilitation workforce. It is not the size of the workforce alone that is critical to improving rehabilitation services. The ways in which the workforce is organised and supported directly impacts the performance of the health system (Chen et al. 2004). Issues including difficulties achieving a suitable mix of skills in health workers, inappropriate distribution of workers, poor working environments and a lack of ongoing training may impact the effectiveness of services (Chen et al. 2004). There is little documented information on how the profession of SLT is organised and supported and the challenges faced by this profession in the delivery of services in SSA.

This paper reports on initial exploratory research into the profession of SLT in SSA. It presents data describing a sample of SLTs in SSA and provides an overview of their employment patterns, work roles and work-related activities undertaken by SLTs. The paper then explores the characteristics of groups of PWCD who receive services provided by the SLTs surveyed. This paper is the second in a series of two. Its companion paper (Wylie et al. 2016) previously described the demographics, education, professional experience and geographical stability of the 33 SLTs who completed the survey.

## Research method and design

Survey research was undertaken to investigate the characteristics, work and employment of SLTs across English-speaking countries in SSA. The methodology for this research has been described in more detail in a previous complementary article (Wylie et al. 2016). The current paper reports on a subset of data from the survey reporting on employment conditions, work roles of the respondents and characteristics of the PWCD to whom they provide rehabilitation services.

### Materials

This research used a purpose-designed survey instrument, developed in line with the process described by Punch (2003). Survey aims were established across five domains: workforce characteristics, SLT education, language and culture, employment and work activities, and continuing education. Survey items were developed, reviewed and revised by the research team who had significant experience as SLTs in Majority World settings. Because of resource limitations, the survey was provided only in English.

Piloting of the survey was undertaken with six SLTs who each had experience working in the Majority World. Participants were requested to provide feedback on the content and structure of the survey, including the readability of each item. The survey was subsequently modified based on feedback from pilot participants.

The final survey contained 186 items and contained both open and closed questions. The survey took between 45 and 60 min to complete, a significant time commitment for participants. In order to improve recruitment and response opportunities, multiple modes of delivery of the survey were offered: online (Survey Monkey), emailed attachment (form) in Microsoft Word, paper copy or via a telephone or callback service.

### Setting

This research was undertaken between April 2012 and March 2013 and sought to recruit SLTs within Anglophone countries of SSA. Twenty Anglophone or partially Anglophone countries were included in the research. South Africa was excluded from the research because of its long history of SLT education (Pillay & Kathard 2015). Selection criteria included self-identifying as an SLT and being resident in one of the target countries for 6 months or longer during the study period.

Because of a lack of regional workplace statistics or listings of professionals, snowball sampling was utilised so that the researchers could draw on local knowledge of SLTs and other networks in the region to locate potential participants. When probability sampling methods are not possible, snowball sampling can be used to access the population under study (Handcock & Gile 2011). The inherent risk of bias of this sampling methodology is acknowledged.

### Procedure

Initial contact was made with a range of potential informants who had contact with or knowledge of communication disability services in target countries. Contacts included SLTs, disability workers, voluntary organisations, professional bodies and academics. Contacts were either known personally to the research team or located through Internet searches and they, in turn, were asked to forward information about the research to potential respondents. General information, survey resources, links for completion of the survey and contact information were provided to potential participants. Paper-based surveys were distributed to eligible SLTs at the East African Conference on Communication Disability in Uganda in 2012.

### Analyses

Raw data from completed surveys were entered into a Microsoft Excel spreadsheet. Text-based categorical responses were numerically coded according to a priori categories where relevant (e.g. identification of African or non-African nationality). Quantitative data were analysed using descriptive statistics. Small sample sizes precluded use of inferential statistical analysis. Data are presented descriptively and must be interpreted with caution.

Open-ended survey responses were analysed using qualitative content analysis as described by Shreier (2012). Following a period of immersion and key word identification, codes were developed inductively from the data and reviewed during first-pass coding; a coding frame was developed and applied to the data from each open question. Data within each code were reviewed to ensure internal consistency. The coding frame and coding were reviewed by a second researcher experienced in qualitative research. Final codes were organised into hierarchies.

## Results

Thirty-three completed surveys were received from SLTs working across nine countries. The demographic mix of respondents is reported in Wylie et al. (2016). Two-thirds of the sample (*n* = 22) identified as African nationals while the remaining one-third (*n* = 11) were non-African nationals, predominantly from European countries.

### Employment and funding sector

All respondents reported currently working, holding at least one job (mean 1.45, range 1–3, mode 1). Thirty-three respondents reported on a total of 44 jobs. Thirty-two jobs were held by 22 African nationality SLTs and 12 jobs were held by non-African nationality SLTs. The majority of respondents held one job only, but one-third (*n* = 11, 33%) of the respondents held more than one job.

The term ‘funding sector’ was used to describe in which sectors SLTs were employed and included government, private and not-for-profit (e.g. non-government organisations and voluntary services) sectors. The largest proportion of SLT jobs were in the private sector (*n* = 20, 45%), followed by the not-for-profit sector (*n* = 15, 34%) and the government sector (*n* = 9, 20%). (Note: Percentages rounded to the nearest percentage point.) Qualitative content analysis confirmed this data. In response to an open-ended question asking respondents to describe each job, SLTs frequently identified their funding sector, including government, private and not-for-profit groups. Descriptions of organisations that were considered not-for-profit included: non-government organisation, not-for-profit, consumer group, international voluntary organisation and Christian mission.

**FIGURE 1:**
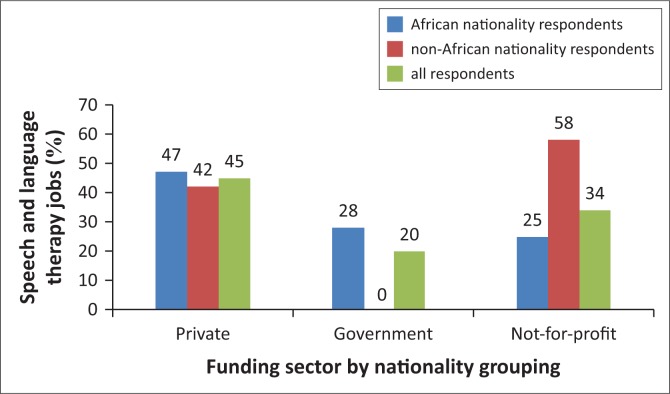
Funding sector of speech and language therapy jobs, by nationality grouping.

When considered by nationality grouping, the largest proportion of jobs held by African nationals (*n* = 15, 47%) were in the private sector, with similar proportions observed in the government (*n* = 9, 28%) and not-for-profit (*n* = 8, 25%) funding sectors. The largest proportion of jobs reported by non-African nationality respondents was in the not-for-profit sector (*n* = 7, 58%), followed by the private sector (*n* = 5, 42%) (Figure 1). No non-African national in this sample reported holding a government-funded job. Small sample size precluded statistical analysis.

### Employment setting

Employment settings indicated the type of service in which the respondent provided services. As an example, a SLT working in a government school would have been considered to be employed *by* the government (funding sector) but employed to offer services *in* schools (employment setting). SLTs were employed in the following settings: non-government organisations (*n* = 15, 34%); private practice (*n* = 13, 30%); health services and hospitals (*n* = 8, 18%); education or schools (*n* = 6, 14%); and tertiary education (*n* = 2, 5%). Results organised by funding sector and employment setting are presented in Table 1.

**TABLE 1:** Speech and language therapy jobs by funding sector and employment setting.

Variables	Health *n* (%)	Education *n* (%)	Tertiary education *n* (%)	NGO *n* (%)	Private practice *n* (%)	Total jobs by funding sector *n* (%)
Private	5 (25)	2 (10)	0 (0)	0 (0)	13 (65)	20 (45)
Government	3 (33)	4 (44)	2 (22)	0 (0)	0 (0)	9 (20)
Not-for-profit	0 (0)	0 (0)	0 (0)	15 (100)	0 (0)	15 (34)

**Total jobs by service setting**	**8 (18)**	**6 (14)**	**2 (5)**	**15 (34)**	**13 (30)**	**44 (100)**

NGO, non-government organisation.

In an open-ended question about job descriptions, respondents described the location of their workplace, including hospitals, schools, special schools, preschools, client homes, private clinics, universities, rehabilitation centres and disability centres.

### Employment patterns

Overall, less than half (18 of 44 jobs, 41%) of all jobs were reported as fulltime. Participants were asked to identify if roles they held were permanent or temporary or to identify if the type of role made this irrelevant (i.e. independent volunteering or self-owned private practice). Overall, of the 44 jobs, 27% (*n* = 12) fell into the not relevant category. The permanency of applicable jobs, by funding sector, is reported in Table 2.

**TABLE 2:** Permanency (applicable roles), overall and by funding sector.

Variables	Permanent *n* (%)	Temporary *n* (%)	Unsure *n* (%)	Total
All applicable jobs	12 (39)	16 (52)	3 (10)	31
**Breakdown by funding sector**				
Private	4 (36)	7 (64)	0 (0)	11
Government	6 (75)	1 (13)	1 (13)	8
Not-for-profit	2 (17)	8 (67)	2 (17)	12

Figures rounded to the nearest percentage point.

Respondents were asked if there was someone else within the organisation doing similar work (i.e. a professional peer in SLT or communication disability). Thirty-eight per cent (*n* = 16) of respondents indicated that they worked alongside a professional peer. The remaining 62% (*n* = 26) indicated that they did not have a professional peer within their work context.

### Recipients of speech and language therapy services

Respondents were asked to indicate if their job was ‘clinical’, involving direct service provision to individuals or groups of people with communication disabilities, or had a ‘non-clinical’ (education, community development or programme) focus, where they did not work directly with PWCD. The following section reports on the 33 ‘clinical’ jobs described in the sample.

#### Geography

SLTs were predominantly located in urban areas including capital cities and other towns and cities (94%), with the majority of SLTs (73%) based in the capital city (Table 3).

**TABLE 3:** Location of speech and language therapists.

Location of SLT	*n* (%)
Capital city	24 (73)
A city, large town or regional centre (not the capital city)	7 (21)
A small town, village or small community	2 (6)
An isolated rural area – but not in a village or community	0 (0)

**Total**	**33 (100)**

SLT, speech and language therapist.

Of the jobs described in this study, two urban-based therapists mentioned visits to rural areas within their job descriptions.

‘I work in a project which visits rural and remote areas on development and integration for people with disabilities, especially communication.’ (ID T001, nongovernmental organisation (NGO)/voluntary)

‘I work all over the country, both in urban and rural environments.’ (ID E002, NGO/voluntary)

Within the qualitative content analysis of job descriptions, there was evidence that PWCD travel to receive services and that travel had an impact on service delivery.

‘Since my clients come from for like 300km I assess, psycho-educate parents and give advice on further management at home, since they can’t stay at the centre.’ (ID PB11, NGO/voluntary)

#### Age

Respondents were asked to rate the frequency in which they worked with people of various ages, using a five-point Likert scale. Responses were aggregated to represent age groups more and less frequently seen and presented in Table 4. More than half of the respondents (*n* = 18, 55%) indicated that they regularly worked with people across the age ranges – from children to adults (sometimes, always or often).

**TABLE 4:** Frequency: age range of speech and language therapy service users.

Age range of service users	Always or often *n* (%)	Sometimes *n* (%)	Occasionally or never *n* (%)
Adults (18+ years)	9 (27)	9 (27)	15 (45)
Adolescents (13–17 years)	10 (30)	11 (33)	12 (36)
School-aged children (6–12 years)	21 (64)	9 (27)	3 (9)
Preschool-aged children (3–5 years)	26 (79)	6 (18)	1 (3)
Babies and infants (0–2 years)	4 (12)	8 (24)	21 (64)

Figures rounded to the nearest percentage point.

Within the open-ended job description, respondents frequently referred to the age of people using SLT services, with a dominance of paediatric clients reported, consistent with the descriptive data.

#### Health-related conditions

Respondents were asked to consider the types of health-related conditions experienced by PWCD accessing SLT services and to rank how commonly people with these conditions accessed services on a five-point Likert scale. The range of conditions included in the survey were based on the observations and clinical experience of the researchers working in the region and explored during pilot testing. Open categories were included to allow respondents to represent conditions that were not covered by predetermined categories in the survey.

Results are ranked in Table 5 indicating the eight most frequently reported conditions of people accessing clinical SLT services and the proportion of SLTs who reported seeing people with this condition ‘always or often’. While the list is unlikely to be comprehensive, because of the diverse range of clients seen by SLTs, it provides an indication of common issues that are experienced by people who seek rehabilitation from SLTs in the sample.

**TABLE 5:** Most frequently reported health-related conditions of people accessing speech and language therapy services.

Health-related conditions	Rank	Proportion of SLTs reporting seeing people with this condition ‘always’ or ‘often’ (%)
Autism spectrum disorders	1	61
Language delay or disorders	2	58
Speech delay or disorders	3	52
Intellectual disabilities	=4	45
Physical disabilities	=4	45
Stroke	6	36
Hearing impairment	=7	27
Stuttering	=7	27

SLTs, speech and language therapists.

Within the open-ended job description, respondents frequently reported seeing people with a variety of different health-related conditions.

‘I see all patient groups as they come, cannot afford to specialize in this kind of work setting where the service is limited.’ (ID E004, NGO/voluntary)

#### Economics of services

Respondents were asked to indicate if direct payment was required for clients to receive SLT services or if services were free at the point of use. Respondents indicated that almost two-thirds (64%) of clients directly paid for SLT services at a level perceived by the therapist to be commensurate with private or commercial rates. The remainder were noted to pay either a small or subsidised fee (18%) or to receive a service free at point of use (18%). Payment levels, by funding sector, are outlined in Table 6.

**TABLE 6:** Perceived rates of payment, by funding sector.

Variables	Private *n* (%)	Government *n* (%)	Not-for-profit *n* (%)	Total *n* (%)
Private level fees	16 (80)	1 (20)	4 (50)	21 (64)
Small fee	3 (15)	1 (20)	2 (25)	6 (18)
Free service	1 (5)	3 (60)	2 (25)	6 (18)

**Total**	**20 (61)**	**5 (15)**	**8 (24)**	**33 (100)**

### Roles of speech and language therapists

A broad range of work-related roles were described by respondents within the open-ended job descriptions. The categories of roles following qualitative content analysis and examples of the types of activities described are provided in Table 7.

**TABLE 7:** Speech and language therapists: Role categories and examples.

SLTs: Role categories	Examples from transcripts of open-ended questions
Therapist	‘We do some therapy and advice for families and train families how to best work with their children.’ (T001, NGO/voluntary)
Team member	‘work closely with OTs, physiotherapists, doctors, audiologists, teachers, educational psychologists and psychologists to ensure a holistic approach to therapy.’ (SM04, private)
Trainer	‘Most of the work is providing training to the carers and parents, and teaching other professionals and volunteers who work with them [and use] the therapeutic strategies.’ (E002, NGO/voluntary)
Administrator	‘I operate the IEPs and co-ordinate the service.’ (PB15, NGO/voluntary)
Facilitator	‘… facilitate three patient/client and carer support/ self-help groups.’ (E001, private)
Advocate	‘… advocating for inclusion in the mainstream schools’ (SM11, private)
Researcher	‘We also do research on communication disorders.’ (T002, government)
Capacity builder	‘Involving the local people in their capacities as much as possible and using approaches that can be supported through the culture and resources locally available.’ (E004, NGO/voluntary)

SLTs, speech and language therapists; NGO, non-government organisation.

Respondent information: (Unique identifier, job funding sector).

### Training others

**FIGURE 2:**
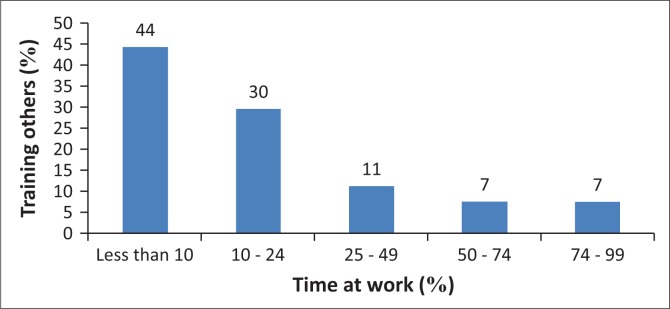
Proportion of time at work spent training others.

Respondents were asked to specify the amount of time they spent training people who were not parents, carers or relatives of clients. Of the 27 responses, the largest proportion (*n* = 12) estimated they spent less than 10% of their time on training others (44%). However, over one-quarter of respondents (*n* = 7) indicated they spent 25% or more of their time training in the workplace (Figure 2). Within the open-ended descriptions of training, participants indicated that they had trained a variety of workers across sectors in the previous 12 months (Table 8).

**TABLE 8:** Examples of groups trained by speech and language therapists.

Sector	Examples of groups trained in the previous 12 months
Health	Nurses, occupational therapists, physiotherapists, psychologists, other therapists (unspecified), doctors, SLT assistants, student nurses, medical students, nursing students, other SLTs, healthcare workers (unspecified).
Disability or community	CBR workers, community workers, rehabilitation technicians, community-based trainers (of the deaf).
Education	Special education teachers, class teachers, preschool teachers, teaching assistants, student teachers, educators (unspecified).
Students	SLT students, students (unspecified), neuropsychology student.
Home	Care workers, support staff.
Other	Workers at other NGOs.

SLT, speech and language therapy; SLTs, speech and language therapists; CBR, community-based rehabilitation; NGO, non-government organisation.

## Ethical considerations

The University of Queensland, Australia, where the first author was enrolled as a doctoral student at the time of the study development, granted ethical approval for this project (reference number 2011-SOMILRE-0018). Informed consent was inferred via survey response. Secure Sockets Layer technology was used to protect online survey data.

## Discussion

This paper reports data from a survey of the work of SLTs in SSA on employment patterns, position funding, characteristics of clients and activities undertaken by SLTs. Overall, private and not-for-profit sectors were the largest employers of SLTs in this sample. African nationality respondents were most frequently employed within the private sector, while non-African nationals were most frequently employed in the not-for-profit sector. As the profession of SLT grows in SSA, not-for-profit organisations may consider recruiting SLTs differently from current models of volunteerism (see Hickey et al. 2012). Rather than importing foreign volunteers, they may have capacity to offer longer term employment to locally based SLTs, which improves potential for service stability and language mix needed for culturally appropriate services (Wylie et al. 2016).

Part-time and temporary roles dominated the sample. Less than half of roles described (41%) were fulltime and less than half of relevant jobs (39%) were reported as permanent. While government jobs were limited, they appeared to offer more stability, with the majority of these roles reported as permanent. Further investigation around whether employment patterns reflect job availability or employee preference is required.

The dominance of part-time and temporary roles prompts questions about service sustainability. Workforce stability has been shown to benefit both the service and the client as it contributes to both improved productivity and skills (Auer, Berg & Coulibaly 2005; Buchan 2010). The high rate of temporary roles, coupled with use of a foreign (non-African) workforce with high turnover (Wylie et al. 2016), indicates that many of the SLT services available may not be stable. Establishment of stable, permanent jobs for the growing local workforce is essential if SLT is to contribute meaningfully to communication disability services in the region.

The availability of stable jobs for SLTs is particularly important with the increasing number of training programmes for SLT in SSA (Wylie et al. 2016). Unless a system of stable jobs is available for SLTs who train in SSA, training programmes may produce graduates who remain unemployed in the field, migrate out of the region to seek stable employment, change profession, or self-employ through private practice.

Governments have a key role to play in disability, including strategy, policy, regulation, resourcing and delivery of rehabilitation services (World Bank & World Health Organization 2011). The lack of SLT roles in the government sector is multifactorial. Historically, because of a range of policy and economic issues, African governments have struggled to employ and retain health workers or invest sufficiently in health infrastructure (Anyangwe & Mtonga 2007). With recent growth in local SLT training (Wylie et al. 2016) increasing the size of the workforce for communication disability rehabilitation, it is unsurprising that positions are lagging behind in government services. If governments in SSA are to ensure a stable, equitable and accessible range of rehabilitation services for communication disability, then it is important to consider how the workforce with skills in communication disability, including SLTs, should be employed and what it will take to drive such a change. Substantial activism may be required to produce policy shifts that prioritise communication disability and establish SLT roles in the government sector (Wickenden 2013; Wylie et al. 2013).

The majority of SLTs (62%) reported that they did not have a SLT colleague within their workplace. As the profession grows, how SLTs are supervised, mentored and supported to enable continuous learning and service quality requires consideration. SLTs reported working in multidisciplinary contexts and reported a wide range of multidisciplinary team members, which may provide some level of generic support in continuous learning.

Lack of professional support and supervision has implications for performance of the workforce (Mathauer & Imhoff 2006; Willis-Shattuck et al. 2008). Potential for career progression into more senior positions has also been shown to impact the motivation of the workforce (Willis-Shattuck et al. 2008). The development of appropriate support systems and career pathways may contribute to a robust and motivated workforce.

SLTs reported engaging in a wide variety of roles within their work, including the provision of direct therapy as well as roles including inclusion support, capacity building and awareness raising, which may reflect a broader role than traditionally seen in SLT (Wickenden 2013). One of the most common categories in the description of work roles was training others. Respondents reported training a diverse range of people across sectors. The sector in which SLTs are employed may influence the type of roles SLTs undertake, including working in areas such as training others, advocacy and awareness raising. Broader roles for SLT may be more constrained in the private sector, where the focus is likely to be on the provision of treatment to individuals.

Training appeared to be a key role for the SLTs in this sample. Widespread training by SLTs in SSA has the potential to support models of rehabilitation that use less specialised service providers, such as mid-tier health workers and CBR workers. The ability of SLTs to train and capacity build with others has been recognised in the literature on development of services for communication disability in the Majority World as essential (Hartley & Wirz 2002; Robinson et al. 2003; Winterton 1998).

Calls to improve the formal training systems for CBR workers (Mannan, MacLachlan & McAuliffe 2012) offer a timely opportunity to reconsider the roles for a profession such as SLT in Majority World contexts, which may differ from those in the Minority World. The dominance of training in job descriptions of SLTs suggests that they may be well placed (and already engaging) in supporting training in communication disability. This is critical in light of the recognition of the lack of training for CBR workers in this area (World Bank & World Health Organization 2011). Using SLTs – who possess specialist skills in communication and swallowing disabilities – to train and support CBR workers, and other health and education workers, has the potential to both increase the coverage of communication disability rehabilitation and improve networks between health-related rehabilitation and CBR (MacLachlan, Mannan & McAuliffe 2011; Mannan et al. 2012; Nganwa et al. 2013; World Bank & World Health Organization 2011).

SLTs reported providing services to a range of people with communication disabilities, including people across the lifespan and with a range of health-related conditions. The focus of services appeared to be predominantly in paediatrics, although respondents reported seeing clients across the age ranges.

The most frequently reported health-related conditions of people seeking SLT services were speech or language delay or disorders, autism spectrum disorder, physical disabilities and intellectual disabilities. The data indicated that many of the SLTs sampled work across a range of areas in a generalist approach, in contrast to countries of the Minority World, where services and systems are well developed and therapists often work in specialised or specific areas of practice.

The generalist nature of the roles described in this sample, with SLTs working in both adult and paediatric populations, and across different health-related conditions, indicate that SLTs require a diverse range of support to maintain and grow skills through continued professional education. Creating alternative networks of support and supervision to meet the needs of SLTs working with diverse caseloads is particularly important as the majority of respondents reported being the only SLT in their workplace.

Services provided by SLTs in the sample were largely provided in urban areas with limited services in rural communities. This is consistent with the maldistribution of the health workforce between rural and urban settings in SSA (Anyangwe & Mtonga 2007) and represents a geographical barrier to SLT service access (Peters et al. 2008).

The cost of services has been shown to be one of a range of barriers to accessing health services (Commission on Social Determinants of Health 2008; Mills et al. 2012; Peters et al. 2008), particularly when repeated or expensive treatments are required (Ansah et al. 2009; James et al. 2006), such as in the case of rehabilitation services.

Almost two-thirds of SLT service users were reported to pay fees commensurate with a private level of service. Payments at this level were reported across private, government and not-for-profit employment sectors; however, the small amount of data was suggestive of government SLT services levying lower direct costs to PWCD. This requires further exploration as costs of services may present an economic barrier to equitable rehabilitation (Peters et al. 2008). While governments in Majority World countries are challenged in financing rehabilitation (World Bank & World Health Organization 2011), creation of SLT roles in sectors with free or low-cost services or models of coverage for PWCD is essential to promote equity of access to rehabilitation. As SLT training programmes develop, if graduate SLTs are siphoned into a system of private practice, possibly contributed to by a lack of government jobs, there is a risk of further promoting inequality, where more affluent urban residents are disproportionately able to access SLT services. Lack of access to rehabilitation may have long-term implications for people with communication disabilities as communication disability may limit the ability of an individual to maximise his or her social and economic independence (Ruben 2000).

## Limitations of the study

This is an initial exploration of the SLT workforce with limited data. Non-probability sampling methods and a small sample size limit the ability to generalise results. Selection bias was likely, as despite the use of multiple response modalities, respondents may have been more likely to respond if they had access to technology or were more connected in the international or local communication disability networks. However, this study offers an early exploration of a sample of the workforce and consideration of important factors of relevance to the profession in the region.

## Conclusion

Speech and language therapy is beginning to grow in SSA with the development of local SLT training programmes (Wylie et al. 2016). It is timely to reflect on how the profession of SLT could and should be organised in SSA, particularly if the aim of expanding the profession is the development of sustainable and equitable communication disability rehabilitation services. This research presents the workforce profile of a sample of SLTs in SSA by describing their employment patterns, selected characteristics of people receiving SLT services and work roles. Services provided by SLTs were provided to PWCD across age ranges, health conditions and settings, with paediatric, urban services commonly reported. Training was a commonly reported role for SLTs in the sample. Consideration of employment, work and service factors has raised a number of issues around how SLTs are employed and work in the region, which may impact service sustainability and accessibility.

While this research has provided initial insights into the role and employment of SLTs and indications of who receives services in the region, much more extensive debate and research is required to consider how the work of SLTs in SSA should be structured and supported. Reconsideration of the role of SLTs is needed to ensure that SLTs contribute to sustainable and accessible communication rehabilitation in a way that is responsive to the rehabilitation needs of people in SSA.
